# Omic studies reveal the pathogenic lipid droplet proteins in non-alcoholic fatty liver disease

**DOI:** 10.1007/s13238-016-0327-9

**Published:** 2016-10-18

**Authors:** Xuelin Zhang, Yang Wang, Pingsheng Liu

**Affiliations:** 1 School of Kinesiology and Health, Capital University of Physical Education and Sports, Beijing, 100191 China; 2 National Laboratory of Biomacromolecules, CAS Center for Excellence in Biomacromolecules, Institute of Biophysics, Chinese Academy of Sciences, Beijing, 100101 China

**Keywords:** non-alcoholic fatty liver disease, lipid droplets, genome-wide association study, proteomics, PNPLA3, 17β-HSD13

## Abstract

Non-alcoholic fatty liver disease (NAFLD) is an epidemic metabolic condition driven by an underlying lipid homeostasis disorder. The lipid droplet (LD), the main organelle involved in neutral lipid storage and hydrolysis, is a potential target for NAFLD therapeutic treatment. In this review, we summarize recent progress elucidating the connections between LD-associated proteins and NAFLD found by genome-wide association studies (GWAS), genomic and proteomic studies. Finally, we discuss a possible mechanism by which the protein 17β-hydroxysteroid dehydrogenase 13 (17β-HSD13) may promote the development of NAFLD.

## INTRODUCTION

Non-alcoholic fatty liver disease (NAFLD) is characterized by a pathological accumulation of triacylglycerol (TAG) in hepatocytes (i.e. hepatic steatosis) without excessive alcohol consumption (Cohen et al., 2011; Hardy et al., 2016). Although the prevalence of NAFLD varies among studies due to the different sensitivities of the instruments used and the ethnic makeup of the populations studied, there is no doubt that the incidence of NAFLD is increasing to epidemic proportions, especially in developing countries. As the incidence of viral infections such as Hepatitis B virus (HBV) and Hepatitis C virus (HCV) is decreasing, NAFLD is becoming the most prevalent liver disease (Wang et al., 2014; Younossi et al., 2015; Rinella and Charlton, 2016). NAFLD encompasses a spectrum of diseases ranging from simple hepatic steatosis, to non-alcoholic steatohepatitis (NASH), to cirrhosis. Severe forms of NAFLD increase the risk of other liver diseases and a portion of patients will ultimately develop hepatocellular carcinoma (HCC) (Cohen et al., 2011; Michelotti et al., 2013). Even though only a small fraction of HCC patients will progress to late stage disease, the increasing prevalence of NAFLD and its sequelae commands attention. In addition to diseases of the liver, NAFLD increases mortality through cardiovascular disease, placing a great burden on public health care systems (Targher et al., 2010; Anstee et al., 2013; Gaggini et al., 2013).

NAFLD is regarded as a manifestation of metabolic syndrome which is influenced by multiple factors (Cohen et al., 2011; Hardy et al., 2016). Genetics play a vital role in the development and progression of the disease, a conclusion which is supported by family cohort studies and ethnic based variations in its incidence and presentation (Anstee and Day, 2013). Increased food intake and a sedentary life style strongly contribute to the establishment of the disease (Anstee et al., 2013). Other metabolic conditions like obesity, insulin resistance, hypertension, and dyslipidemia are also risk factors associated with NAFLD (Smith and Adams, 2011). There are no medications specifically intended to treat NAFLD, but drugs for related conditions including insulin sensitizing agents, antioxidants and lipid-lowering agents are often used in its treatment (Musso et al., 2012; Dowman et al., 2011). It is still unknown why a fraction of NAFLD patients develop NASH or cirrhosis. Therefore, it is vital to uncover the pathogenic mechanisms driving the development of this disease and its sequelae.

Hydrophobic neutral lipids (primarily TAG and cholesteryl ester (CE) in eukaryotic organisms) are mainly stored in an organelle termed the lipid droplet (LD) (Murphy, 2001). The LD consists of a neutral lipid core covered by a monolayer of phospholipids and associated proteins (Tauchi-Sato et al., 2002). Rather than inert energy reservoirs, LDs are actively associated with other organelles and play vital roles in lipid metabolism, membrane trafficking and signal transduction (Martin and Parton, 2006; Zehmer et al., 2009). Dysfunction in this organelle can result in disorders of lipid metabolism, which makes the LD a promising target for research into the pathogenic mechanisms of NAFLD (Greenberg et al., 2011; Krahmer and Farese, 2013; Carr and Ahima, 2016).

The protein complement on LDs which drive the organelle’s functions also likely underlie the metabolic pathology that leads to NAFLD. The Perilipin family (Plin1–5) are the major LD proteins in mammalian cells (Kimmel et al., 2010). Plin1 is hardly detected in normal liver LDs, but its expression is prominently up-regulated in fatty liver LDs (Straub et al., 2008; Fujii et al., 2009). Similarly, Plin2 expression is increased in NAFLD, both in human and rodents, and is associated with oxidative damage (Fujii et al., 2009). Knocking out Plin5 in mice results in reduced hepatic lipid content due to elevated lipolysis and fatty acid oxidation, but also induces lipotoxic injury (Wang et al., 2015). The cell death-inducing DFFA-like effector (CIDE) proteins are located on LDs and the endoplasmic reticulum (ER) and are also involved in fatty liver progression. *Cidea* and *Fsp27* (*Cidec*) mRNA levels are significantly increased in fatty liver dystrophic mice (Hall et al., 2010). The CIDEB protein is predominately expressed in the liver. *Cideb*-null mice are resistant to diet-induced obesity and show decreased lipogenesis and increased fatty acid oxidation (Li et al., 2007).

Lipolysis-related proteins, like CGI-58, might also be involved in the pathogenesis of fatty liver. The liver specific ablation of CGI-58 causes NASH and fibrosis (Guo et al., 2013), which is consistent with the clinical presentation of the CGI-58 mutation induced Chanarin-Dorfman syndrome (Srinivasan et al., 2004; Ronchetti et al., 2008). Hypoxia-inducible gene 2 (Hig2), which is primarily localized on LDs, impairs TAG hydrolysis in liver, thereby promoting hepatic lipid accumulation (DiStefano et al., 2015). The loss of LDs and retinoid content in hepatic stellate cell (HSC) is a hallmark of NASH. Blockage of lipolysis by lipases like ATGL in HSC may prevent or alleviate the development of fibrosis (Blaner et al., 2009).

In this review, we focus on LD proteins that may be involved in the pathogenesis of NAFLD as determined by Genome-wide Association Studies (GWAS), genomic and proteomic research. As the mechanisms are still unknown, we also propose a hypothesis based on recent studies.

## GWAS reveal pathogenic proteins on lipid droplets like PNPLA3

With the completion of the Human Genome Project, scientists focused on variable regions in the genome, especially single nucleotide polymorphisms (SNPs) associated with diseases. Under the HapMap project, haplotype maps were established using tag SNPs. Disease associations with novel genes can be established by GWAS using a non-candidate driven method (Hardy and Singleton, 2009; Manolio, 2010). GWAS is especially useful in parsing complicated traits not caused by a single genetic mutation but by complex contributions from environmental factors and polygenic components, which describes NAFLD (Anstee and Day, 2013). GWAS studies focused on NAFLD were summarized by Anstee and Day (2013), Sookoian and Pirola (2015). The SNPs involved in NAFLD include *PNPLA3* (rs738409), *GCKR* (rs780094), *NCAN* (rs2228603), *LYPLAL1* (rs12137855), *PPP1R3B* (rs4240624), *CPN1-ERLIN1-CHUK* gene cluster, as well as others. These gene polymorphisms may influence NAFLD via hepatic carbohydrate and lipid metabolism, protein modification or signaling pathways (Anstee and Day, 2013; Sookoian and Pirola, 2015).

Patatin-like phospholipid domain containing protein 3 (PNPLA3) is a LD-associated protein that is also distributed on other membranes (He et al., 2010). A GWAS performed as part of the Dallas Heart Study was the first to find an association between *PNPLA3* and NAFLD (Romeo et al., 2008). PNPLA3 has the strongest linkage disequilibrium of all hits and has consistently been identified in multiple GWAS using different diagnostic criteria (Anstee and Day, 2013). Moreover, it has been demonstrated by numerous independent candidate-driven and histology-based studies that the I148M polymorphism is associated with and increased risk of NASH, fibrosis, and NAFLD-related HCC (Kotronen et al., 2009; Sookoian et al., 2009; Rotman et al., 2010; Dongiovanni et al., 2013). PNPLA3-I148M may promote liver injury as detected by elevated serum ALT levels (Kollerits et al., 2010). PNPLA3-I148M is proportionally overrepresented in the Hispanic population (0.49 in Hispanics, 0.23 in European-Americans and 0.17 in African-Americans), which may partially explain the higher susceptibility Hispanic people to NAFLD (Romeo et al., 2008).

The *PNPLA3* gene is located on chromosome 22 and encodes a 53-kDa protein with 481 amino acids. PNPLA3 in humans is predominantly expressed in the liver, with substantially lower expression level in skin and adipose tissue (Huang et al., 2010). PNPLA3 belongs to the patatin-like phospholipid domain containing (PNPLA) protein family. It is most closely related to PNPLA2, which is also known as adipose triglyceride lipase (ATGL), the major cellular TAG lipase. PNPLA3 has TAG hydrolase activity which is markedly reduced in the I148M variant. Structural analysis of PNPLA3 has demonstrated that the mutation does not affect the catalytic center but rather the groove of the substrate binding domain. Therefore the I148M substitution possibly blocks access of substrate to the catalytic site (Huang et al., 2011).

Much work has been conducted on the physiological and pathological functions of PNPLA3 and the I148M variant. However, the mechanism underlying its association with disease remains enigmatic. PNPLA3 expression is very low in a fasted state, and is strongly induced with feeding. The expression of PNPLA3 is up-regulated by insulin through LXR/RXR signaling to SREBP-1c. This synthesis of fatty acids, which is stimulated by SREBP-1c, increases the half-life of PNPLA3 (Huang et al., 2010). These results indicate that PNPLA3 is regulated by both lipid and carbohydrate metabolism in response to the nutrition environment (Huang et al., 2010). However, the disease associated genetic variants of PNPLA3 are not linked with risk factors such as insulin sensitivity or body mass index (BMI) and do not affect related metabolic syndromes such as dyslipidemia or type 2 diabetes (Romeo et al., 2008; Speliotes et al., 2010). Besides TAG lipase activity, PNPLA3 is also reported as possessing lysophosphatic acid acyltransferase (LPAAT) activity *in vitro* and retinyl-palmitate lipase activity in human HSCs (Kumari et al., 2012; Pirazzi et al., 2014). Since the development of NASH is accompanied by a loss of retinoid content in HSCs, PNPLA3 may play a role in the hepatic inflammatory process.

Although TAG hydrolysis activity of PNPLA3-I148M is decreased, the association between the genetic variant and NAFLD seems not to be due to the loss of function. The *Pnpla3* deletion in mice does not affect liver TAG content even under high-sucrose or high-fat diet conditions (Chen et al., 2010; Basantani et al., 2011) and human PNPLA3 overexpression in mice does not decrease liver steatosis (Li et al., 2012). On the contrary, overexpression of *PNPLA3-I148M* in mice leads to liver steatosis with elevated lipogenesis and impaired TAG hydrolysis (Li et al., 2012). TAG content and LD size are increased in high-sucrose diet fed *Pnpla3-I148M* knock-in mice without a significant change of lipogenic genes. However, CGI-58, which facilitates ATGL in hydrolyzing TAG, is increased dramatically on LDs (Smagris et al., 2015). This result suggests that PNPLA3 may alter lipolysis, not by the hydrolysis activity itself, but by inhibition of other lipases. Moreover, PNPLA3-I148M affects VLDL secretion in rat hepatoma McA-RH7777 cells, possibly due to a decreased ability to mobilize TAG in LDs (Pirazzi et al., 2012). PNPLA3-I148M may also enhance TAG synthesis by elevating LPAAT activity (Kumari et al., 2012). A disadvantage of the mouse model in the study of the I148M variant function is the different tissue distribution, compared with humans. PNPLA3 is primarily expressed in liver in humans, while it is mainly expressed in adipose tissue in the mouse (Huang et al., 2010; Hoekstra et al., 2010). Further studies will be required to determine how PNPLA3 is associated with NASH and fibrosis.

There are 21 other SNPs in PNPLA3 identified in the Dallas study which are potentially linked to NAFLD. Among them is PNPLA3-S47A, in which the catalytic serine is substituted by alanine, which results in decreased lipolytic activity and increased hepatic lipid content (He et al., 2010; Huang et al., 2011; Smagris et al., 2015). Variant S543I is more common in African-Americans than in European-Americans or Hispanics and is associated with decreased lipid content in the liver (Romeo et al., 2008). The *434K* allele attenuates PNPLA3 protein expression by decreasing PNPLA3 mRNA levels and also attenuates the association of I148M with liver damage (Donati et al., 2016).

## Proteomic studies on isolated lipid droplets reveal 17β-HSD13 is involved in THE pathogenesis of NAFLD

Metabolic syndromes like NAFLD are affected not only by genomic alterations but also by complicated factors like environment. As a result, genomic analysis alone is insufficient as it is removed from the actual expression of proteins (Gregorich and Ge, 2014). Chronic diseases always manifest as continuous processes with evolving stages. There are often biomarkers which shift with the development of the disease and identification of diagnostic biomarkers is especially important for detecting early stage disease. Proteomics can be used to identify alterations in protein expression and post translational modifications characteristic of diseases during different stages of their development at the level of tissue, cell, organelle or other subcellular structures. In addition, proteomic analyses of disease states can also identify novel drug targets for drug development (Hanash, 2003). Proteomics can also help reveal protein complexes and signaling networks (Gregorich and Ge, 2014). Tremendous improvements in the sensitivity and throughput of modern mass spectrum technologies have led to a dramatic expansion in protein research in the past decade. Many studies of NAFLD have been conducted at various disease stages, revealing 34 candidate biomarkers in proteomic studies of liver and serum (Lim et al., 2014; Ladaru et al., 2016).

Dozens of proteomic analyses have been performed on isolated LDs in many types of cells and tissues from various organisms (Yang et al., 2012), including liver tissue and hepatocytes from humans and rodents (Yang et al., 2012; Crunk et al., 2013; Su et al., 2014; Khan et al., 2015). Structural proteins, like those of the perilipin family, are prominent in most LD proteomes. Other LD proteins can be categorized into groups including proteins of lipid synthesis and hydrolysis, membrane trafficking, and cell signaling. The presence of these functional classes of proteins on LDs speaks to their central role in lipid metabolism. Apolipoproteins are present on liver LDs suggesting an association between LDs and lipid secretion. Moreover, large numbers of mitochondrial and ER proteins are also found in the liver LD proteome. This suggests the existence of close physical and functional interactions among these organelles, likely involving fatty acid oxidation and steroid metabolism (Crunk et al., 2013; Su et al., 2014; Khan et al., 2015).

Comparative LD proteomic studies reveal that 54 proteins are up-regulated and 35 proteins down-regulated in human livers with simple hepatic steatosis. The up-regulated proteins are involved in the metabolism of retinol, linoleic acid, xenobiotics and drugs (Su et al., 2014). The liver LD proteome of mice with diet-induced hepatic steatosis has also quantitatively analyzed using iTRAQ. As with human liver, the up-regulated proteins included those involved in fatty acid catabolism and xenobiotic metabolism, and also included some ribosomal and ER proteins. Among the down-regulated proteins were the liver X receptor, retinoid X receptor, and proteins involved in glucose metabolism. (Khan et al., 2015). These results provide new insights into the metabolic pathways of hepatic steatosis and may point to possible drug targets for the treatment of NAFLD.

Of the proteins of potential clinical interest identified in proteomic studies, 17β-hydroxysteroid dehydrogenase 13 (17β-HSD13) is possibly the most important. A comparative, quantitative proteomic study in humans detected a dramatic elevation of 17β-HSD13 in patients with NAFLD, compared with healthy controls (Su et al., 2014). Another independent study confirmed this result and also found a slight upregulation of 17β-HSD13 in patients with NASH without fatty liver (Kampf et al., 2014). In a study of fasted and refed mice, 17β-HSD13 was increased markedly on hepatic LDs of mice in the high-fat diet group compared with the low fat group (Crunk et al., 2013).

In parallel, a GWAS study revealed that an intergenic SNP (rs6834314) near HSD17B13 (encoding 17β-HSD13) and MAPK10 (encoding mitogen-activated protein kinase 10, MAPK10) is strongly associated with the concentration of plasma alanine transaminase (ALT) (*P* = 3.1 × 10^−9^), the main marker of hepatocellular injury (possibly representing fatty liver disease) (Chambers et al., 2011). Overexpression of 17β-HSD13 in a mouse hepatocyte cell line induced liver steatosis and lipid accumulation. It also lead to increased expression of proteins involved in lipid synthesis such as mature SREBP-1 and FAS, suggesting that 17β-HSD13 is implicated in NAFLD development by promoting lipogenesis (Su et al., 2014). 17β-HSD13 is expressed primarily in the liver, with far less found in the gastrointestinal tract, muscle, spleen and uterus, making 17β-HSD13 an excellent potential therapeutic target for treating fatty liver disease (Horiguchi et al., 2008).

17β-HSD13 was first cloned from a human liver cDNA library in 2007 and was named short-chain dehydrogenase/reductase 9 (SCDR9) (Liu et al., 2007). Now the protein has been grouped with the 17β-hydroxysteroid dehydrogenase (17β-HSD) family, which plays a key role in the final step of estrogen and androgen steroid metabolism. As with the other members of 17β-HSD family (except 17β-HSD5), 17β-HSD13 contains a NAD(P)^+^/NAD(P)H binding domain (TGxxxGxG) and an enzymatic activation site (YxxxK) at the N-terminus. The 17β-HSDs are regarded as potential therapeutic targets for diseases such as breast cancer, endometriosis, osteoporosis, prostate cancer and even Alzheimer’s disease. All 17β-HSDs can modify the keto and hydroxy groups of steroids at the position C17 *in vitro*. However, the real physiological function of 17β-HSD13 *in vivo* is still unknown (Moeller and Adamski, 2006; Marchais-Oberwinkler et al., 2011).

17β-HSD13 is located at the same locus with 17β-HSD11 on chromosome 4q22. Moreover, 17β-HSD13 shares a high sequence similarity (65% identity and 78% similarity) with 17β-HSD11 (Liu et al., 2007). The 1–28 amino acid region of the N-terminus of 17β-HSD11 is necessary and sufficient to target the protein to ER and LD, while similar targeting activity is located in the first 35 amino acids of the N-terminus of 17β-HSD13 (Horiguchi et al., 2008). These results indicate that these two proteins may share features of their function and regulation.

However, the mRNA expression of 17β-HSD11 is strongly induced in liver and intestine by PPARα agonist Wy14643, while 17β-HSD13 is not induced. On the contrary, the 17β-HSD13 expression level is significantly elevated in PPARα knockout mice, which suggests that PPARα may suppress the expression of 17β-HSD13 (Horiguchi et al., 2008). 17β-HSD13 has two putative C/EBP binding sites while 17β-HSD11 has four. Expression of both proteins was induced by overexpression of C/EBPβ in HepG2 cells while only 17β-HSD11 expression was induced by overexpression of C/EBPα (Rotinen et al., 2010). These results suggest 17β-HSD13 and 17β-HSD11 may play different roles under physiological and pathological conditions in liver.

## A role for 17β-HSD13 on lipid droplets in the pathogensis of NAFLD?

Sex hormones play important roles in maintaining energy homeostasis and imbalances in their levels can lead to metabolic syndromes like type 2 diabetes (Varlamov et al., 2014). Epidemiological studies have revealed a higher prevalence of NAFLD in men than in women. However, the incidence of NAFLD increases rapidly in postmenopausal women, ultimately erasing the gender difference. This implicates estrogen in regulating hepatic lipid metabolism (Hashimoto and Tokushige, 2011; Brady, 2015; Shen and Shi, 2015). The metabolic protective effect of estrogen in preventing liver steatosis has also been detected in animal models. Female mice treated with ovariectomy (OVX), and a resulting depletion of the majority of circulating estrogens, have increased fat accumulation in the liver than the pair-fed sham operation mice (Shen and Shi, 2015).

Tamoxifen is an estrogen inhibitor that is widely used in the treatment of hormone-sensitive breast cancer. Approximately 43% of the patients treated with this drug develop hepatic steatosis, likely due, primarily, to increased *de novo* fatty acid synthesis in the liver (Cole et al., 2010). All subtypes of estrogen receptors are expressed in hepatocytes and estrogen receptor-α predominantates (Shen and Shi, 2015). Treatment with the estrogen receptor-α agonist propyl pyrazole triol (PPT) decreases fat content in the liver. Estrogen receptor-α knockout mice display liver steatosis with lipid biosynthesis genes, including SREBP-1c, up-regulated and the lipid transport genes downregulated (Shen and Shi, 2015; Barros and Gustafsson, 2011).

Unlike what has been seen with estrogens, studies examining androgen signaling have found complex effects with inconsistent results and significant variation between genders (Ma et al., 2014). An inverse relationship between circulating testosterone levels and hepatic steatosis has been found in men (Volzke et al., 2010; Kim et al., 2012). However, other studies found that anabolic-androgenic steroids induce hepatotoxicity and result in NAFLD (Schwingel et al., 2011; Awai et al., 2014). These conflicting results may arise from the steroid dose (ranging from physiological to supra-physiological levels) or the type or ratio of androgens studied.

A low plasma level of dehydroepiandrosterone sulfate (DHEA-S), a kind of proandrogen, is associated with advanced NAFLD (Charlton et al., 2008) Dehydroepiandrosterone (DHEA) supplementation of a high-fat plus fructose diet reduced the induction of SREBP-1c and insulin resistance in mice, and protected against steatosis (Aragno et al., 2009). In females, an abnormally high level of androgens is associated with polycystic ovary syndrome (PCOS) with a high risk of NAFLD (Brzozowska et al., 2009; Kelley et al., 2014). Male, but not female, androgen receptor (AR) knockout mice fed a high-fat diet develop hepatic steatosis and insulin resistance with an increased expression of lipogenic genes and a decreased expression of fatty acid oxidation genes (Lin et al., 2008). These results indicate that androgen deficiency is associated with hepatic lipid deposition and the AR may have greater impact on lipid homeostasis in males than in females.

A significant fraction of estrogens and androgens are synthesized locally inside of target peripheral tissue cells, a process described as intracrine regulation. Almost all sex steroids in postmenopausal women and half of androgens in adult men are synthesized in peripheral intracrine tissues (Labrie et al., 2000). Tumor cells adapt the intracrine system to produce high level of estrogens or androgens to stimulate cell proliferation in sex steroid sensitive diseases like breast and prostate cancer (McNamara and Sasano, 2015; Mostaghel, 2013).

Circulating DHEA and DHEA-S originating from the adrenal gland are the major substrates for intracrine sex hormone synthesis and the conversion involves enzymes belonging to 3β-HSD, 17β-HSD, 5α-reductase and aromatase families (Labrie, 2015). Members of the 17β-HSD family are the key enzymes required for synthesizing all active estrogens and androgens. The 17β-HSD proteins are distributed broadly in most tissues where they play a vital role in intracrine sex steroid homeostais (Luu-The and Labrie, 2010). Hepatic progenitor cells in humans and hepatocytes in rats have the ability to activate TGF-β signaling by intracrine signaling, which supports that liver is an intracrine organ (Gressner et al., 2008; Ding et al., 2013). Since more than half of all known types of 17β-HSDs have been found in the liver (Moeller and Adamski, 2009), it is reasonable to speculate that the liver can also generate intracrine sex steroid signaling.

**Figure 1:**
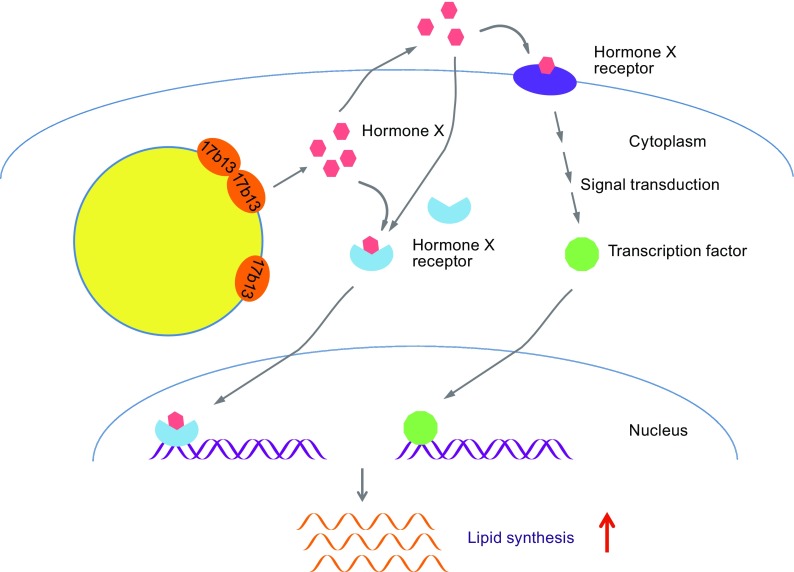
**Hypothetical mechanism of 17β-HSD13-mediated lipid accumulations**. Increased 17β-HSD13 on LDs produces hormone X that binds to a cytosolic receptor by intracrine regulation. The hormone X may also be secreted to extracellular space and bind to a membrane-bound receptor or crosses the plasma membrane and binds to a cytosolic receptor by autocrine or paracrine regulation. The cytosolic ligand-bound receptors thus enter the nucleus and bind to regulate elements. The activated membrane-bound receptors may transduce signals to promote transcription factors enter nucleus. Hence, the expression of genes related to lipid synthesis are eventually up-regulated leading to lipid accumulation and LD expansion in hepatic cells

We propose a hypothesis to explain how increased 17β-HSD13 promotes lipid synthesis and expands LDs in hepatocytes (Fig. 1). Excessive expression of 17β-HSD13 proteins results in the production of increased sex steroid hormone intermediates (or possible other, unknown hormones). The locally produced hormones drive intracrine signaling and may also be secreted to the extracellular space signaling to local tissues in a paracrine fashion. This enhanced signaling drives the expression of lipid synthetic genes, driving fat accumulation in LDs.

The mechanisms involved may be more complicated. 17β-HSD enzymes interconvert estrogens in both directions. Therefore, 17β-HSD13 might convert E2 to E1, thereby reducing the E2 concentration in the cellular microenvironment, resulting in a decrease in metabolic protection afforded by E2 and activating lipid anabolic genes. Besides steroid metabolism, some subtypes of 17β-HSDs also play roles in fatty acid elongation and β-oxidation, synthesis of prostaglandin and retinol metabolism. These other activities of 17β-HSD13 may influence fatty liver development via distinct metabolic pathways. In addition, 17β-HSD13 is also present as a major protein on hepatic LDs. Thus it may also act as a structural protein, protecting lipids from hydrolysis by preventing lipases from gaining access to the interior lipids. The mechanisms regulating 17β-HSD13 and its involvement in the progression of NAFLD still need to be elucidated.

## CONCLUSION

The LD is the central organelle regulating lipid homeostasis and disorders of LD-associated proteins induce metabolic diseases like NAFLD. GWAS studies reveal that SNPs linked with PNPLA3 are strongly associated with NAFLD, especially the *PNPLA3-I148M* variant. PNPLA3-I148M seems to be a loss-of-function mutation, but its pathogenic effect appears to be mediated through a gain in function resulting in a suppression of lipase activity. The 17β-HSD13 enzyme was identified by a comparative proteomic study of patients with normal and simple hepatic steatosis liver. Increased 17β-HSD13 on LDs greatly elevates lipid content in both hepatocytes and mouse liver. Since 17β-HSD family proteins catalyze the key step in sex hormone synthesis, we speculate that 17β-HSD13 may promote lipid synthesis by producing hormones which regulate lipogenic gene expression. However, the detailed underlying mechanisms by which LDs and LD-associated proteins induce NAFLD remain to be elucidated.
